# Optimized Refeeding vs. Standard Care in Malnourished Older Hospitalized Patients: A Prospective, Non-Randomized Cluster-Controlled Study in Geriatric Acute Care

**DOI:** 10.3390/jcm12237274

**Published:** 2023-11-24

**Authors:** Maryam Pourhassan, Diana Daubert, Thea Laurentius, Rainer Wirth

**Affiliations:** 1 Department of Geriatric Medicine, Marien Hospital Herne, Ruhr-Universität Bochum, 44625 Herne, Germany; diana.daubert@elisabethgruppe.de (D.D.); rainer.wirth@elisabethgruppe.de (R.W.); 2 Universitätsklinikum Aachen—Standort Franziskus Anstalt des öffentlichen Rechts (AöR), 52074 Aachen, Germany; tlaurentius@ukaachen.de

**Keywords:** malnutrition, management, nutritional support, nutritional therapy, mortality, older hospitalized patients

## Abstract

Malnutrition is a prevalent geriatric syndrome with adverse health outcomes. This study aimed to assess the effectiveness of an optimized protocol for treatment of malnutrition in older hospitalized patients. We conducted a prospective, non-randomized cluster-controlled study with 156 malnourished patients in the intervention and 73 in the control group, determined using the Mini Nutritional Assessment-Short-Form. The intervention group received individualized nutritional care, including electrolyte and micronutrients monitoring, while the control received standard care. We primarily focused on complications such as infections, falls, unplanned hospital readmissions, and mortality, and secondarily focused on functional status and mobility improvements. Post-discharge follow-ups occurred at 3 and 6 months. Our findings demonstrated that the intervention group (age 82.3 ± 7.5 y, 69% female), exhibited greater previous weight loss (11.5 kg vs. 4.7 kg), more cognitive impairment and a longer hospital stay (19 days vs. 15 days). Binary logistic regression showed no difference in primary endpoint outcomes between groups during hospitalization. At 3- and 6-month follow-ups, the control group exhibited fewer adverse outcomes, particularly falls and readmissions. Both groups showed in-hospital functional improvements, but only controls maintained post-discharge mobility gains. The study concludes that the nutritional intervention did not outperform standard care, potentially due to study limitations and high-quality standard care in control group geriatric departments.

## 1. Introduction

Malnutrition, defined as a state of nutrient deficiency or excess due to imbalances between nutrient intake and requirements, is a major geriatric syndrome characterized by a high prevalence and a multifactorial etiology [1]. Malnutrition is linked with an elevated risk of adverse outcomes, including infections, falls, sarcopenia, compromised quality of life, extended duration of hospital stays, and increased rates of mortality and morbidity [2,3]. Previous studies have indicated that the prevalence of malnutrition in older patients can reach up to 50% [4,5]. However, it is important to note that these prevalence estimates exhibit considerable variability, depending on the population under investigation, the healthcare context, and the method implemented for the malnutrition assessment [6,7,8].

Clinical guidelines endorse systematic malnutrition screenings for elderly patients, followed by individualized nutritional assessments. Positive screenings necessitate personalized nutritional support, encompassing specific dietary counselling, oral supplements, fortified foods and, as necessary, enteral or parenteral feeding [9]. This standardized nutritional strategy is key to early intervention, which is critical in both preventing and treating malnutrition in this vulnerable population [9]. The early detection and management of malnutrition in older patients may substantially reduce healthcare utilization, decrease the risk of mortality and enhance quality of life [10,11]. Nevertheless, the treatment and management of malnutrition in older patients presents a complex challenge, requiring the collaborative efforts of a multidisciplinary healthcare team. Consequently, to achieve meaningful progress, the treatment of malnutrition demands the implementation of a comprehensive intervention strategy. This approach should encompass multiple facets of care, targeting improvements in nutritional status, ensuring adequate energy intake and enhancing overall clinical outcomes [12]. Several meta-analyses have demonstrated that providing oral nutritional supplements (ONS) is effective in older patients and has the potential to decrease mortality, morbidity, complications, and unplanned hospital-readmissions, and increase muscle strength [13,14].

In line with existing evidence, we hypothesize that the efficacy of nutritional intervention may be significantly enhanced through the execution of a comprehensive and more personalized treatment strategy and by considering further aspects. Notably, this approach should account for potential complications such as refeeding syndrome and individual nutritional demands, address micronutrient deficiencies, and provide personalized nutritional counseling and education. Additionally, integrating transitional care could ensure consistent nutritional management, even after hospital discharge. Therefore, we aimed to evaluate the effectiveness of an optimized protocol for the treatment of malnutrition by measuring the differences in clinical outcome between those receiving the optimized intervention and those subjected to nutritional standard care throughout the hospital stay and subsequent follow-up periods.

## 2. Methods

### 2.1. Study Design and Setting

This is a prospective, non-randomized, cluster-controlled intervention study involving older hospitalized patients in geriatric acute care hospital wards who were consecutively admitted between May 2019 and January 2022. The intervention group, consisting of 156 patients, was recruited from the geriatric acute care unit of Marien Hospital Herne, university hospital of Ruhr-University Bochum, Germany. Simultaneously, a control group of 150 patients was planned to be recruited from three other geriatric departments in Germany; Geriatric department of the hospital in Schwelm, Center for Geriatrics and Gerontology in Hamburg and Department of Geriatric Medicine of the university Aachen. The control group was offered standard care without the help of a dedicated nutrition support team. The rationale for this multicenter control group was to minimize the influence of local preference regarding patient’s care.

### 2.2. Intervention Protocol

In addition to standard care, patients in the intervention group underwent various interventions, as detailed in Table 1.

### 2.3. Study Procedure

Throughout the study’s duration, all patients were screened against the set inclusion and exclusion criteria during their inpatient admission. The inclusion and exclusion criteria were uniformly applied to both the intervention and control groups.

### 2.4. Inclusion and Exclusion Criteria

Inclusion criteria for recruitment were patients aged 70 years or above who were identified as malnourished. Malnutrition was determined using the Mini Nutritional Assessment-Short Form (MNA-SF) score of less than 8, or if the patient experienced more than 10% weight loss from their initial body weight within a 6-month timeframe or less. Additional criteria included the need for nutritional support, an anticipated hospital stay of at least 14 days, and the capacity and willingness to provide informed consent. Exclusion criteria were severe dementia, severe cognitive impairments of other origin, severe dysphagia, severe depression, expected requirement of tube feeding for more than two weeks and a palliative situation.

### 2.5. Geriatric Assessment

A geriatric assessment was carried out upon admission in both intervention and control groups as follows: The MNA-SF [16] was utilized to assess patients’ nutritional status, encompassing factors such as dietary intake, three-month weight loss, mobility, psychological stress, acute diseases, neuropsychological issues, and Body Mass Index (BMI). Based on their scores, patients were classified as normal nutritional status (12–14 points), at risk of malnutrition (8–11 points), or malnourished (0–7 points). Cognitive function was assessed differently in the two groups, according to the local standard procedures, using the MoCA [17] for the intervention group and the MMSE [18] for the control group. Patients scoring less than 26 on MoCA and below 24 on MMSE were identified as having cognitive impairment. The control group’s MoCA scores were predicted using Bergeron et al. conversion table [19]. The evaluation of depressive symptoms also differed between the groups. For the intervention group, the Depression in Old Age Scale (DIA-S) was used [20], while the Geriatric Depression Scale (GDS-15) [21] was applied for the control group. A DIA-S score ranging between 4–10 and a GDS-15 score above 6 were indicative of probable depression. In addition, data regarding prior unintentional weight loss and the corresponding time period were collected through interviews with the patients and if incompetent their relatives, Furthermore, patients were asked about their discharge destination, which could include home, short- or long-term care facilities, rehabilitation clinics, or transference to another hospital.

### 2.6. Functional and Mobility Status

Functional and mobility status was evaluated in both intervention and control groups. Patients’ ability to carry out self-care tasks was measured using the Barthel Index (BI) [22] at two timepoints: upon admission and at discharge. This German version of the BI rates scores between 0 and 100, with a score of 100 indicating complete independence in performing all daily living activities. Mobility status was assessed using the Parker Mobility Score [23] at four points: hospital admission, discharge, and at 3- and 6-month follow-up. This score relies on three mobility-related questions, each scoring between 0 and 3. The total score, derived from the sum of three distinct mobility assessments (ability to move around the house, to leave the house, and to go shopping), ranges from 0 to 9. The three situations are rated as follows: no difficulty (3 points), use of aid (2 points), need for human assistance (1 point), or inability to perform the task (0 points). A maximum score of 9 reflects optimal mobility.

### 2.7. Follow Up Examination

During follow-up, we investigated the incidence of adverse events, including instances of infections, mobility assessment, unexpected hospital re-admissions and cases of mortality. For these particular aspects, except the mobility assessment, we utilized a structured, but non-validated approach. However, the mobility assessment was evaluated using the Parker mobility score, which is validated as a tool to be applied by telephone interview [24,25,26]. Post-discharge follow-up evaluations were conducted at 3 and 6 months via standardized brief telephone interviews with either the patients themselves or their proxies.

### 2.8. Primary and Secondary Endpoints

The effectiveness of our comprehensive and optimized protocol for the treatment of malnutrition was tested by evaluating the following outcomes:

Primary endpoint: We examined the occurrence of complications, which included infections, falls, unplanned hospital readmissions and mortality. These were recorded during the hospital stay, and then followed up at three and six months after discharge. The combined primary endpoint was then calculated as the sum of the occurrences of these specified complications. If a patient experienced even a single occurrence of these complications, they were defined as having a positive combined primary endpoint.

Secondary endpoint: Our evaluation extended to the treatment and rehabilitation success, determined by comparing the functional status, as measured by BI and defined by any increase in BI, between admission and discharge. In addition, sustained treatment and rehabilitation success was measured by evaluating the patients’ mobility, as evaluated by parker mobility score, from the time of hospital admission, through discharge, and at three and six months after discharge.

## 3. Data Analysis

The statistical analysis was conducted using SPSS Statistics for Windows software (Version 29.0, IBM Corp, Armonk, NY, USA). Anticipating a substantial average increase of 24 points in the Barthel index for the intervention group (and 19 in the control group), and considering a realistically high standard deviation of ± 17 points, we determined that a sample size of n = 143 in a 1:1 design would provide a statistical power of 0.8 and a type I error rate of 0.05 (http://PowerAndSampleSize.com, accessed on 15 November 2017). Continuous variables were represented by means and standard deviations (SDs) for normally distributed data, and by medians with interquartile ranges (IQR) for data that did not adhere to a normal distribution. Absolute numbers and relative frequencies (%) were used to express categorical variables. Differences within patients’ variables and between the intervention and control groups were evaluated using an unpaired *t*-test for normally distributed data, the Mann–Whitney U test for non-parametric, non-normally distributed variables, and the Chi-square test for categorical variables. Furthermore, changes in mobility status across four timepoints (from admission to the 6-month follow-up) were examined using the Friedman ANOVA test in both groups. We employed a binary logistic regression analysis to investigate the potential differences in primary endpoints between the intervention and control groups. The combined primary endpoint was determined as a sum of the occurrences of complications, namely infections, falls, unplanned hospital readmissions, and mortality. If a patient experienced even a single occurrence of these complications, they were defined as having a positive combined primary endpoint. Accordingly, in this regression model, the dependent variable was this combined endpoint, while the independent variable was the intervention type (either intervention or control). Notably, covariates that exhibited significant differences between the groups at admission were included to adjust the analysis (refer to the Results section, binary logistic regression analysis). This approach allowed us to discern the effect of the intervention while controlling for potential baseline differences between the two groups. The derived model provided odds ratios (OR) accompanied by their 95% confidence intervals (CI) and their associated *p*-values for the combined endpoint. Additionally, we evaluated the goodness-of-fit for each logistic regression model using the Hosmer–Lemeshow test, ensuring the models’ appropriateness for the observed data. A *p*-value of less than 0.05 was set as the threshold for statistical significance.

## 4. Results

### 4.1. Participant Flow: Recruitment to Follow-Up Analysis

**Figure 1:**
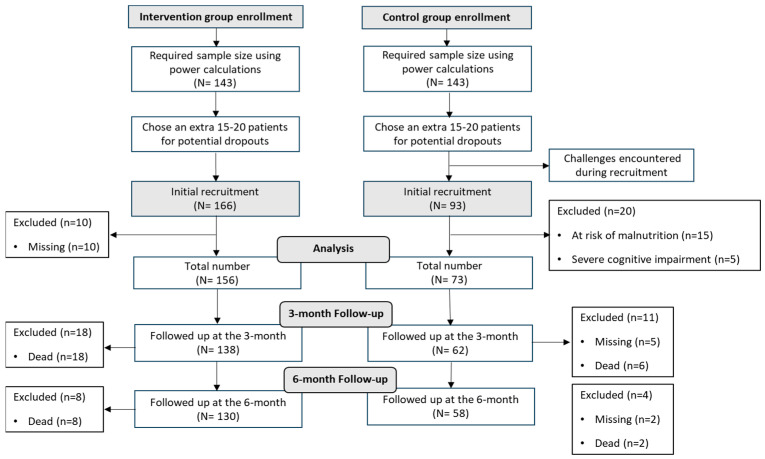
Flowchart illustrating patient recruitment, exclusion, and follow-up stages for both intervention and control groups in the study.

A flowchart illustrating patient recruitment, exclusion, and follow-up stages for both intervention and control groups in the study is presented in Figure 1. In brief, the initial recruitment for the intervention group comprised 166 patients. However, 10 of these patients were subsequently excluded due to incomplete geriatric assessments, missing prior weight loss history, or unavailable electrolyte and micronutrients measurements because of an inability to conduct a blood test. This resulted in a final number of 156 patients entering the intervention group for the study. Within this group, 18 patients had died by the 3-month follow-up and an additional 8 had passed away by the 6-month follow-up.

On the other hand, the control group started with an initial recruitment of 93 patients. Out of these, 20 patients were excluded: 15 due to being at risk of malnutrition and 5 due to severe cognitive impairment. This left 73 patients as the final number for the control group. Within this group, at the 3-month follow-up, there were 5 patients who could not be located for assessment and 6 patients who had deceased. By the 6-month follow-up, an additional 2 patients were lost to follow-up, and 2 more had passed away.

### 4.2. Baseline Characteristics of Participants

Table 2 shows the baseline characteristics of the study participants within both the intervention and control group.

A majority of participants across both cohorts were female. No significant differences were noted between the two groups in terms of gender distribution, age, and BMI. The age range was 70–100 years in the intervention group, while it ranged from 70 to 93 years in the control group. In each group, all patients were malnourished, with a median MNA-SF score of six. Among those in the intervention group, eight patients were identified as at risk of malnutrition. However, these patients had experienced weight loss exceeding 10% of their initial body weight within a six-month period or less, thus classifying them as malnourished according to the inclusion criteria.

### 4.3. Differences between Intervention and Control Group

A substantially higher proportion of participants in the intervention group (97%) reported previous weight loss compared to control group (64%). Further, the mean previous weight loss in the intervention group was significantly higher (11.5 kg) in contrast to the control group (4.7 kg). In addition, there was a more pronounced impairment of cognitive function in the intervention than the control group and the length of stay in the hospital was longer for the intervention group, with a median of 19 days, compared to a median of 15 days in the control group. Nearly all patients in the intervention group received nutritional therapy, compared to 75% of patients in control group (<0.001).

### 4.4. Binary Logistic Regression Analysis of Combined Primary Endpoints

We performed a binary logistic regression model (Table 3) to analyze the differences in the combined primary endpoint (infections, falls, unplanned hospital readmissions and mortality) between intervention and control groups, while adjusting for several potential confounding factors: weight loss (kg), cognitive impairment and length of hospitalization. Our analysis revealed no significant differences in the combined primary endpoint between the intervention and control group at hospital discharge. However, at the 3-month and 6-month follow-up assessments, there was a significantly lower frequency of the combined primary endpoint within the control group when compared to the intervention group. Furthermore, the Hosmer–Lemeshow test was employed to evaluate the goodness-of-fit of our model. The results were as follows: at hospital discharge (*p* = 0.896), at 3 months post-discharge (*p* = 0.484), and at 6 months post-discharge (*p* = 0.652), indicating a good fit between the observed outcomes and the model predictions at all timepoints. In addition, none of the potential confounding factors included in our model reached statistical significance at any timepoint. Notably, the differences in combined primary endpoint between the two groups were predominantly driven by falls (*p* = 0.001) and unplanned hospitalization (*p* = 0.001) at the 3-month follow-up and by falls (*p* = 0.001), unplanned hospitalization (*p* = 0.022) and mortality (*p* = 0.007) at the 6-month follow-up.

### 4.5. Functional and Mobility Status over Time

The functional and mobility status of the study participants at hospital admission, discharge and throughout the follow-up period, are presented in Table 4.

Within group: A significant improvement in the Barthel Index was noted during hospitalization for both intervention and control groups (*p* < 0.001 for both). Moreover, an observable improvement in the parker mobility score from admission to discharge was present in the intervention group, although this did not represent a statistically significant progression over time. The parker mobility score returned to baseline levels during the 3 (*p* = 0.835) and 6-month (*p* = 0.535) follow-up periods in the intervention group. Conversely, the control group exhibited a significant, continuous improvement in the parker mobility score over time.

Between group: At the time of admission, a minor difference was observed in the Barthel Index between the intervention and control groups, although this difference did not reach statistical significance upon discharge (Table 4). Similarly, no substantial variances were detected in the parker mobility score between both groups at the point of admission. Further, the control group exhibited a significantly elevated parker mobility score at the time of discharge, a trend that continued during the 3- and 6-month follow-up periods when compared to the intervention group.

## 5. Discussion

Our study aimed to evaluate the effectiveness of a comprehensive, personalized nutritional intervention on malnourished older hospitalized patients in comparison to geriatric standard care over a period of 6 months post-discharge and contrasted a combined endpoint of mortality and other complications during this time. Despite the robust protocol of our intervention strategy, our findings revealed an unexpectedly higher complication rate in the intervention group compared to the control group, not at hospital discharge, but during follow-up.

The results of our study contradict several meta-analyses that have demonstrated the effectiveness of providing oral nutritional supplements in older patients, resulting in a decrease in mortality, morbidity, complications and unplanned hospital-readmissions [14,27,28]. In a comprehensive review investigating the effectiveness of dietary supplementation in improving nutritional status and clinical outcomes, data from 62 trials, comprising a total of 10,187 randomized participants (≥65 years), were analyzed [29]. The interventions in these trials extended up to 18 months. The findings revealed that there was no significant reduction in mortality rates within the supplemented groups when compared to control groups across 42 of these trials. However, in contrast to our results, when focusing on malnourished participants, mortality rates were significantly reduced in the supplemented groups of the meta-analysis. Further, a decline in the risk of complications was observed in 24 trials. Nevertheless, only a minority of trials reported functional benefits from supplementation. Additionally, no significant effect on the length of hospital stay was evident across 12 trials. In addition, our study results are in contrast with the findings of the EFFORT trial [30], which showed the effectiveness of individualized nutritional support in improving clinical outcomes for medical inpatients at nutritional risk. However, it is important to acknowledge that significant distinctions between two studies include the strict randomization and the high discrepancy in sample size, with the EFFORT trial involving a larger population (2088 participants) compared to our study (229 participants). Nevertheless, probably most importantly, the EFFORT trial primarily focused on medical inpatients, while our study specifically targeted older hospitalized adults in a geriatric department. The robust management of malnutrition in the geriatric setting, which appears to be well-established and effective in many departments, may contribute to the lack of observed intervention efficacy in our study.

Although the combined primary endpoint in our study did not significantly vary between the intervention and control groups during hospitalization, the 3- and 6-month follow-up assessments exhibited a consistently lower rate of these complications within the control group relative to the intervention group. Interestingly, the significant differences were largely driven by factors such as falls, unplanned hospitalizations, and mortality rates. The unexpected results can potentially be attributed to a variety of factors. Firstly, the differences between the intervention and control group may be influenced by the smaller sample size of the control group. The initial recruitment target was 150 participants; however, due to unforeseen challenges during the recruitment process, the final number was limited to 73 patients. This discrepancy might have contributed to an imbalance between the groups, potentially skewing the comparative results, providing an overrepresentation of the combined primary endpoint in the intervention group. Moreover, it is worth noting that two out of the three control centers encountered challenges with sufficient patient recruitment, with 64% of the control group patients originating from one center. This uneven distribution might have introduced bias into the data and potentially limited the scope of our findings, as the results were predominantly driven by a single center’s patient population. Therefore, the ideal of achieving a truly balanced multi-center comparison group was not accomplished in this study.

Second, the intervention group was characterized by a higher level of frailty, substantial multimorbidity, significantly more pronounced cognitive impairment, more pronounced weight loss, and an extended hospital stay in comparison to the control group, i.e., the intervention group suffered from more severe morbidity. These characteristics potentially underlie the elevated complication rates observed within this group, as these patients, due to their compromised health status, inherently bore an elevated risk of adverse outcomes. This underlying vulnerability could have undermined the effectiveness of the nutritional intervention, thereby contributing to a higher rate of complications. It is noteworthy that, while our regression analysis adjusted for key confounding factors such as previous weight loss, duration of hospitalization and cognitive impairment, these factors failed to reach statistical significance in association to the combined primary endpoint at both the 3-month and 6-month follow-up. However, the absence of statistical significance does not necessarily prevent clinical relevance. In a real-world clinical setting, these factors may significantly affect patient outcomes, particularly in older populations. Nevertheless, these relationships might not be adequately captured within the confines of a regression model for several reasons, including the inherent complexity of these relationships, the presence of variable interactions, or the influence of other unmeasured confounding variables on the outcomes. Furthermore, we implemented measures to manage the risk of RFS and micronutrient deficiencies within the intervention group. However, despite these measures, the observed outcomes did not improve. Nonetheless, this finding aligns with the results of a meta-analysis that concluded that the addition of micronutrient supplementation to oral nutritional supplements does not lead to improved outcomes [27].

In addition, the unexpected findings could be influenced by the fact that a significant proportion of patients in the control group (75%) also received nutritional therapy (as standard care), although less than the intervention group (99%). However, the provision of nutritional therapy for 75% of patients is indicative of a high level of nutritional standard care, which addressed the nutritional needs of most patients, minimizing the relative impact of our comprehensive intervention. Indeed, this high standard could present challenges in introducing optimized interventions that could yield more significant improvements. Moreover, the potential of our comprehensive intervention might not have been fully achieved, for several reasons. For instance, while the intervention group received individualized nutritional counseling, the absence of home visits might have impacted the overall effectiveness of the transitional care plan, as there was no direct monitoring of their nutritional intake post-discharge. Although the dietary plans were created to each participant’s food preferences and offered prior to discharge, the lack of oversight and follow-up may have hampered their adherence to the plan, potentially attenuating the intervention’s impact.

### Limitations and Strengths of the Study

Our findings must be interpreted in light of certain limitations: the smaller-than-intended control group, potential center-specific bias, the higher baseline morbidity of the intervention group and the absence of home visitations to monitor post-discharge adherence to dietary recommendations. We postulate that these limiting factors contributed to a notable imbalance between the intervention and control cohorts, leading to the emergence of unexpected results. Nevertheless, when team knowledge is an integral part of the intervention, executing a randomized controlled trial becomes inherently complex. This consideration necessitated the selection of a non-randomized controlled study design in our research methodology. However, it is important to underscore the inherent strengths of our research. A standout feature of our study was the thorough monitoring of key biochemical parameters. Specifically, upon admission, and subsequently on the 1st, 2nd, 3rd, 5th, 7th, and 10th days post-initiation of nutritional therapy, we tracked these levels. Through this continuous monitoring, we were able to identify the risk of RFS promptly and implemented the appropriate prevention strategies. Additionally, within 24 h post-admission, we conducted a detailed analysis of a complete panel of micronutrients, ensuring that our nutritional assessments were both timely and comprehensive. A further strength was our approach to nutritional counseling. This was adapted for each patient, based on data from indirect calorimetry—a method infrequently utilized in geriatric patients, thus offering a unique perspective in our analysis. Consequently, our findings contribute valuable insights to the field of geriatric nutrition and highlight the potential of personalized, comprehensive intervention. Our study results show that individualization and transitional care needs to be more elaborated than that in our study. However, it is clear that further research is needed with an improved study design, such as more balanced group sizes and composition, home visits, post-discharge monitoring and better implementation of intervention strategies. This would enhance the reliability of future studies and our understanding of effective interventions for malnutrition in older hospitalized patients.

## 6. Conclusions

The complex and individualized nutritional intervention failed to improve outcomes in comparison to a control group, probably due to the several study limitations observed in our study. Nevertheless, it offered significant insights into the multifaceted challenges of managing malnutrition in older hospitalized patients. Further research is essential in order to optimize nutritional care in geriatric departments.

## Figures and Tables

**Table 1:** Overview of interventions implemented for patients in the intervention group.

Individualized nutritional support	Based on assessments and specific patient needs, the patients received individualized nutrition counselling delivered by a trained dietitian. This individualized approach ensured that each patient’s unique dietary needs and circumstances were taken into account, aiming to optimize their nutritional status during their hospital stay.
Electrolyte Monitoring	For the first week of admission, a systematic monitoring of potential electrolyte insufficiency, specifically focusing on phosphate and magnesium, was conducted to prevent and detect early signs of RFS. When the risk of Refeeding Syndrome (RFS) was detected, we implemented appropriate prevention strategies (e.g., thiamin was administered promptly) and performed early treatment if a RFS was detected. It is worth mentioning that details about RFS, including its epidemiology and pathophysiology, incidence and prevalence in this population, are published elsewhere [15].
Micronutrient Deficiency Screening	We conducted a comprehensive examination and analysis of a set of important micro-nutrients, including but not limited to vitamins A, B1, B6, B12, C, D, E, H, and K, along with folic acid, iron, ferritin, transferrin, zinc, copper, and selenium.
Micronutrient Supplementation	Based on individual micronutrient status assessments, patients were provided with appropriate single or multi-micronutrient supplementation as needed.
Body Composition Measurement	Muscle mass, fat-free mass, fat mass, and intra/extracellular water were evaluated using Bioelectric Impedance Analysis (BIA).
Resting Energy Expenditure (REE) Measurement	REE was measured using Indirect Calorimetry (IC), and Total Energy Expenditure (TEE) was assessed.
Transitional Nutritional Care	Upon discharge, all patients in the intervention group were provided with an informational brochure containing post-discharge nutritional recommendations

**Table 2:** Characteristics of the study groups at baseline.

	Intervention Group (n = 156)	Control Group (n = 73)	*p* Value
Gender (n, %)			
Females	108 (69)	44 (60)	0.230
Males	48 (31)	29 (40)	
Age (y)	82.3 ± 7.5	81.5 ± 6.0	0.391
Height (m)	1.64 ± 0.08	1.66 ± 0.11	0.153
Body weight (kg)	62.8 ± 13.9	67.8 ± 17.7	0.035
BMI (kg/m^2^)	23.1 ± 4.5	24.4 ± 5.4	0.074
Geriatric assessments			
MNA-SF, Median (IQR)	6 (5–7)	6 (5–7)	0.440
At risk of malnutrition (n, %)	8 (5)	-	
Malnourished (n, %)	148 (95)	73 (100)	
DIA-S, Median (IQR)	3 (1–5) ^a^	-	
GDS-15, Median (IQR)	-	2 (1–4) ^b^	
MoCA, Median (IQR)	17 (14–21) ^a^	20 (15–23) ^c^	<0.001
MMSE, Median (IQR)	-	26 (23–28) ^b^	
Previous weight loss			
Yes (n, %)	151 (97)	47 (64)	<0.001
No (n, %)	4 (3)	26 (36)	
Previous weight loss (kg)	11.5 ± 7.1	4.7 ± 6.5	<0.001
Duration of previous weight loss (months)	8.1 ± 7.1	2.2 ± 2.8	
Nutrition therapy (n, %)			
Yes	153 (99)	54 (75)	<0.001
No	1 (1)	18 (25)	
Discharge to (n, %)			
Home	122 (80)	47 (65)	0.003
Short term-care	23 (15)	13 (18)	
Long term-care	3 (2)	4 (6)	
Rehabilitation clinic	1 (1)	7 (10)	
Another hospital	5 (3)	1 (1)	
Length of stay in days, Median (IQR)	19 (14–21)	15 (14–19)	0.024

MNA-SF, Mini Nutritional Assessment Short Form; DIA-S, Depression in Old Age Scale; MoCA, Montreal Cognitive Assessment; GDS-15, Geriatric Depression Scale; MMSE, Mini Mental State Examination. Values are given as number (%), mean ± SD or median (IQR, interquartile range). ^a^ DIA-S and MoCA were measured in intervention group. ^b^ GDS-15 and MMSE were measured in control group. ^c^ MoCA was predicted in control group using MMSE-MoCA conversion table by Bergeron et al.

**Table 3:** Binary logistic regression analysis of combined primary endpoint of the study groups during hospital stay and during follow-up.

^a^ Combined Primary Endpoint	Intervention Group	Control Group	Binary Logistic Regression Analysis				
			B	Exp (B)	*p* Value	95% CI	
At hospital (n, %)							
Yes	53 (34)	18 (25)	−0.737	0.479	0.116	0.191	1.198
No	103 (66)	55 (75)					
3 months after discharge (n, %)							
Yes	95 (61)	21 (31)	−1.529	0.217	<0.001	0.088	0.536
No	61 (39)	47 (69)					
6 months after discharge (n, %)							
Yes	116 (74)	37 (51)	−1.099	0.333	0.011	0.142	0.779
No	40 (26)	36 (49)					

^a^ The combined primary endpoint was calculated as a sum of the occurrences of the complications such as infections, falls, unplanned hospital readmissions and mortality. If a patient experienced even a single occurrence of these complications, they were defined as having a positive combined primary endpoint. CI, 95% confidence intervals.

**Table 4:** Functional and mobility status stratified by the study groups at admission, discharge and during follow-up.

	Intervention Group	Control Group	*p* Value (Between Group)
Barthel Index, Median (IQR)			
Admission	45 (40–55)	45 (30–55)	0.041
Discharge	70 (55–80)	65 (45–75)	0.235
*p* value (within group)	<0.001	<0.001	
Parker mobility score, Median (IQR)			
Admission	3 (2–5)	4 (1–6)	0.184
Discharge	4 (2–5)	5 (3–6)	0.016
3 months follow-up	3 (2–5)	5 (4–7)	<0.001
6 months follow-up	3 (2–5)	6 (5–7)	<0.001
*p* value (within group)	<0.001	<0.001	

## Data Availability

Additional data are available from the corresponding author upon reasonable request.
